# An exploratory prospective phase II study of preoperative neoadjuvant bevacizumab and temozolomide for newly diagnosed glioblastoma

**DOI:** 10.1007/s11060-023-04544-8

**Published:** 2024-01-31

**Authors:** Toshihide Tanaka, Ryota Tamura, Jun Takei, Yukina Morimoto, Akihiko Teshigawara, Yohei Yamamoto, Ryotaro Imai, Yuki Kuranari, Kyoichi Tohmoto, Yuzuru Hasegawa, Yasuharu Akasaki, Yuichi Murayama, Keisuke Miyake, Hikaru Sasaki

**Affiliations:** 1 Department of Neurosurgery, Jikei University School of Medicine Kashiwa-shi Hospital, 163-1 Kashiwa-shi, Kashiwa, Chiba, 277-8567 Japan; 2 Department of Neurosurgery, Keio University School of Medicine, 35 Shinano-machi, Shijuku-ku, Tokyo, 160-8582 Japan; 3 Department of Neurosurgery, Jikei University School of Medicine, 3-25-8 Nishi-Shinbashi, Minato-ku, Tokyo, 105-8461 Japan; 4 Department of Neurosurgery, Jikei University School of Medicine Daisan Hospital, 4-11-1 Izumi-honcho, Komae-shi, Tokyo, 201-8601 Japan; 5 Department of Neurosurgery, Kagawa University Graduate School of Medicine, 1750-1 Ikedo, Miki-cho, Kida-gun, Kagawa, 761-0793 Japan; 6 Department of Neurosurgery, Tokyo Dental College Ichikawa General Hospital, 5-11-13 Sugano, Ichikawa-shi, Chiba, 272-8513 Japan

**Keywords:** Angiogenesis, Expanding perifocal edema, Exploratory phase II study, Glioblastoma, Neoadjuvant bevacizumab, Wound healing complications

## Abstract

**Purpose:**

This multi-institutional phase I/II study was conducted to confirm the safety and explore the clinical utility of preoperative Bevacizumab (Bev) for newly diagnosed glioblastoma (GB).

**Methods:**

Patients were enrolled based on magnetic resonance imaging (MRI) findings typically suggestive of GB. Preoperative Bev and temozolomide (TMZ) were administered at doses of 10 mg/kg on day 0 and 150 mg/m^2^ on days 1–5, respectively. Surgical resection was performed between days 21 and 30, inclusive. The safety and efficacy were evaluated in a total of 15 cases by progression-free survival (PFS), changes in tumor volume, Karnofsky Performance Scale (KPS) and Mini-Mental State Examination (MMSE) scores after preoperative therapy.

**Results:**

Tumor resection was performed on a mean of day 23.7. Pathological diagnosis was GB, isocitrate dehydrogenase (IDH)-wildtype in 14 cases and GB, IDH-mutant in 1 case. Severe adverse events possibly related to preoperative Bev and TMZ were observed in 2 of the 15 patients, as wound infection and postoperative hematoma and thrombocytopenia. KPS and MMSE scores were significantly improved with preoperative therapy. Tumor volume was decreased in all but one case on T1-weighted imaging with contrast-enhancement (T1CE) and in all cases on fluid-attenuated inversion recovery, with mean volume decrease rates of 36.2% and 54.0%, respectively. Median PFS and overall survival were 9.5 months and 16.5 months, respectively.

**Conclusion:**

Preoperative Bev and TMZ is safe as long as the instructions are followed. The strategy might be useful for GB in some patients, not only reducing tumor burden, but also improving patient KPS preoperatively.

Trial Registration Number: UMIN000025579, jRCT1031180233 https://jrct.niph.go.jp/latest-detail/jRCT1031180233. Registration Date: Jan. 16, 2017

**Supplementary Information:**

The online version contains supplementary material available at 10.1007/s11060-023-04544-8.

## Introduction

Bevacizumab (Bev) is a monoclonal antibody against VEGF, blocking endothelial proliferation and vascular permeability, which could reduce both tumor volume and perifocal edema in GB. These effects might contribute to lessen the surgical burden with decreases in vascularity and improved brain swelling. Indeed, preoperative neoadjuvant Bev therapy has been shown to decrease tumor size and increase pathological response in several cancers, with mostly acceptable toxicity [1–4].

Based on previous randomized clinical trials [5, 6], Bev has been approved for both newly diagnosed and recurrent high-grade gliomas in Japan. In the course of daily Bev use, we have encountered several cases in which Bev was used in the neoadjuvant setting either for safer surgical resection or as a consequence of the clinical course. In those cases, including 8 cases in which tumor resection was performed within 28 days after last Bev administration, we realized significant reductions in tumor vascularity and brain swelling without particular adverse events [7].

Based on our previous experience with preoperative use of Bev, the present multicenter prospective phase I/II study of preoperative Bev and TMZ was conducted. The aim of this study was to confirm safety and feasibility and to explore the clinical utility of preoperative Bev for newly diagnosed GB.

## Methods

### Patient registration

Enrollment was conducted at three university hospitals between January 2017 and November 2021, with the goal of enrolling 15 patients. To avoid possible incorrect diagnosis due to lack of histological verification, patients with lesions with ring-like enhancement on contrast administration with extensive perifocal edema were selected. Selection and exclusion criteria are shown in Table 1.

**Table 1:** Eligibility, exclusion criteria, assessment, and endpoints

Selection criteria
Based on preoperative MRI, typically as ring-like enhancement on contrast administration with perifocal edema
Other clinical/laboratory data, and clinical course
Age 18–75 years
Eastern Cooperative Oncology Group PS;0–2 (with PS 3 allowed if due to neurological deficit caused by brain tumor)
Adequate hematologic, cardiac, pulmonary, renal, and hepatic function
Absolute neutrophil count ≥1,500/μL
Platelets ≥75,000/μL
Hemoglobin 8.0 ≥g/dL
Serum creatinine ≤1.5 mg/dL
AST/ALT ≤3 × upper limit of normal
Total bilirubin ≤2.0 mg/dL

Exclusion criteria
An infectious disease needing systemic treatment
History of cancer within 5 years
History of radiotherapy to the skull
Uncontrolled hypertension
Serious cardiac disease
Any symptomatic thromboembolic event within 6 months
Broncho-pulmonary/symptomatic cerebral hemorrhage within 6 months
Unhealed wound or bone fracture, active gastrointestinal ulcer, pulmonary fibrosis/interstitial pneumonia
Hemoptysis ≥ CTCAE grade 2 within 28 days
Bleeding diathesis or coagulation disorder (prophylactic antiplatelet/anticoagulant drug allowed when PT-INR is 1/5–2.5)
Pregnant or breastfeeding woman, or history of allergic events to gadolinium
Patients with the possibility of metastatic brain tumors or primary central nervous system lymphoma or abscess were also excluded based on serological tumor cell markers, chest x-ray, body CT, and brain MRI including DWI (robust high intensity signal)

Assessment	
Radiological response	within 14 days before registration (baseline) within 7 days before resection within 72 h after resection 1 month after resction every 2 months thereafter
KPS	within 5 days before registration; within 7 days before resection; 1 month after resection; and every 2 months thereafter
MMSE	within 5 days before registration and within 7 days before resection (2–3 weeks after Bev administration)
Endpoints	
Primary endpoint	PFS as assessed by RANO criteria
Secondary endpoint	Safety
	response to preoperative Bev and TMZ
	2-year OS
Subsidiary endpoint	changes in KPS and MMSE by preoperative Bev and TMZ

*AST* aspartate aminotransferase; *ALT* alanine aminotransferase; *Bev* bevacizumab; *CT* computed tomography; *CTCAE* common terminology criteria for adverse events; *DWI* diffusion-weighted imaging; *KPS* karnofsky performance scale; *MMSE* mini-mental state examination; *MRI* magnetic resonance imaging; *OS* overall survival; *PFS* progression-free survival; *PS* performance status; *RANO* response assessment in neuro-oncology; *TMZ* temozolomide

All patients provided written informed consent. The protocol was approved by the ethics committees, and the study was conducted in accordance with the Declaration of Helsinki.

### Treatment

Patients were treated preoperatively with Bev at a dose of 10 mg/kg on day 0 and TMZ at a dose of 150 mg/m^2^ on days 1–5. Surgical resection was performed between days 21 and 30 with careful evaluation of the extent of tumor bulk based on preoperative MRI including T1-weighted MRI with contrast-enhancement (T1CE) and diffusion-weighted imaging (DWI). Following resection, radiotherapy and concurrent and adjuvant TMZ were prescribed according to the published protocol no later than 4 weeks after resection [8]. Radiotherapy was planned as per daily practice of each institution based on baseline MRI before preoperative Bev and TMZ. Adjuvant TMZ was intended to be administered for more than five courses. The extent of resection was evaluated by T1-weighted MRI with contrast-enhancement (T1CE) within 72 h after resection, and classified as gross total resection (GTR; with no residual tumors), subtotal resection (STR; removal of ≥ 90%), or partial resection (PR; removal of < 90%). After a case of surgical wound infection (Case 2), the protocol was amended with the addition of the following instructions regarding wound handling; (1) subdermal sutures should be firmly placed; (2) suture removal is to be performed ≥ 14 days after surgery; and (3) use of carmustine wafer is to be withheld during the resection surgery.

### Assessment and follow-up

Brain MRI was performed at the prespecified timing (Table 1 and Supplementary Fig. 1). MRI sequence included T1CE, T2-weighted imaging, fluid-attenuated inversion recovery (FLAIR), and DWI. In addition, apparent diffusion coefficients (ADCs) were evaluated in all cases by verification of region of interests encompassing the entire tumor volume, whereas perfusion MRI was performed in several cases. Karnofsky Performance Scale (KPS) and the Mini-Mental State Examination (MMSE) were evaluated before and after Bev administration (Table 1).

### Endpoints

The primary endpoint was progression-free survival (PFS) as assessed by Response Assessment in Neuro-Oncology criteria [9]. Secondary endpoints were safety, response to preoperative Bev and TMZ, and 2-year overall survival (OS). Subsidiary endpoints included changes in KPS and MMSE by preoperative Bev and TMZ. Survival was calculated from day 0 of preoperative Bev and TMZ. All enrolled patients were followed until death or 2 years after treatment initiation. Response was assessed by changes in tumor volume as the sum of products of perpendicular diameters of all slices of all measurable lesions on T1CE and FLAIR images. Adverse events (AEs) were evaluated using CTCAE version 4.0. The initial safety evaluation was made for the initial 7 cases. For these 7 cases, AEs within 3 months of resection surgery were evaluated, with the study to be stopped and not proceed beyond Case 7 in the event of more than two non-hematological severe AEs (SAEs) possibly related to preoperative Bev and TMZ, or multiple cases that did not undergo resection surgery during the prespecified period due to reasons possibly related to the preoperative Bev and TMZ. Efficacy was evaluated in total of 15 cases by intention to treat. Both of safety and efficacy was to be evaluated by the investigators and a data and safety monitoring committee (DSMC). PFS was compared with representative historical data for newly diagnosed GB including EORTC26981/22981/NCIC CE.3, and JCOG0911 [8–10]. Associations between response to preoperative Bev and TMZ and patient survival were also investigated on an exploratory basis according to T1CE, FLAIR and ADCs for each.

### Statistical analyses

Continuous variables are provided as the mean ± standard deviation or median, and categorical variables as numbers and percentages. The paired t test was used for comparisons of continuous variables between groups. The log-rank test was used to compare survival differences following the Kaplan–Meier method. All p-values were two-sided with the significance level set to < 0.05. Statistical analyses were performed using STATA 14 software (Stata Corp LP, College Station, TX).

## Results

### Patient characteristics

Patient characteristics are summarized in Table 2 and Supplementary Table 1. Mean age was 62.2 years (range, 36–74 years). All tumors showed strong, ring-like enhancement on T1CE with extensive perifocal edema in accordance with the eligibility criteria (Supplementary Fig. 1). Pathological diagnoses of tumors resected following the single dose of Bev and 5 days of TMZ were GB, IDH-wild type in 14 cases and GB, IDH-mutant in 1 case, according to the WHO 2016 criteria [11]. Mean MIB-1 index was 31.6% (range, 10–80%) (Table 2).

**Table 2:** Characteristics of patients

characteritics (n = 15)			%
Age		62.2 ± 10.8	
		(36–74)	
Sex	Man	9	60
	woman	6	40
Side	Left	10	66.7
	Right	5	33.3
Location	frontal	3	20.0
	temporal	8	53.3
	parietal	3	20.0
	occipital	1	6.7
KPS	before neoBev	83.3 ± 15.4	
		(60–100)	
	after neoBev	94.7 ± 7.4	
		(80–100)	
MMSE	before neoBev	21.3 ± 8.7	
		(6–30)	
	after neoBev	27.5 ± 4.2	
		(18–30)	
MIB-1 index (%)		31.6 ± 18.6	
		(10–80)	
Interval between neoBev to surgery (days)		23.7 ± 2.8	
		(21–29)	
Response to preoperative Bev and TMZ	partial response	4	26.6
	Stable	10	66.7
	progressive disease	1	6.7
Extent of resection	GTR	11	73.3
	STR	3	20.0
	PR	1	6.7
Surgical modalities	Gliadel	1	6.7
	awake surgery	2	13.3
Salvage surgery		6	40.0
Pattern of recurrence	Local	9	60.0
	distant	3	20.0
	local and distant	2	13.3
mPFS (months)		9.5	
mOS (months)		16.5	

*Bev* bevacizumab; *GTR* gross total resection; *KPS* karnofsky performance scale; *MMSE* mini-mental state examination; *mOS* median overall survival; *mPFS* median progression-free survival; *PR* partial resection; *STR* subtotal resection; *TMZ* temozolomide

### Initial safety evaluation

Safety evaluations for the initial 7 cases demonstrated 1 non-hematological SAE within 3 months of resection surgery possibly related to the preoperative therapy, as grade 3 wound infection in Case 2 in which a carmustine wafer had been placed. Neither cancelation nor postponement of resection surgery was required for any of the 7 cases and resection surgery was performed as scheduled between days 21 and 30. The DSMC therefore judged the study protocol as tolerable and approved enrollment of the additional 8 cases with a protocol amendment regarding wound handling (see the Methods section).

### Tumor resection

In all 15 cases, tumor resection was performed between days 21 and 30 (mean, day 23.7; range, day 21–29). Tumors appeared light brownish in coloration, with less vascularity and improved brain swelling, rather than the greyish to brownish with prominent hypervascularity seen in usual glioblastoma surgery. Extent of resection was GTR in 11 cases, STR in 3, and PR in 1. Although no difficulty with hemostasis was encountered during resection, postoperative hemorrhage in the resection cavity was identified in Case 8.

Intraoperative fluorescence diagnosis with 5-ALA was used in all patients and was effective, suggesting that protoporphyrin IX fluorescence was undiminished by the preoperative therapy (Fig. 3I).

One patient underwent trephination surgery under local anesthesia, since the intensive care unit was unavailable due to the COVID-19 pandemic (Fig. 3).

Radiotherapy and concurrent TMZ was commenced after suture removal, and no later than 4 weeks after resection.

### Responses to preoperative bevacizumab and temozolomide

**Fig. 1:**
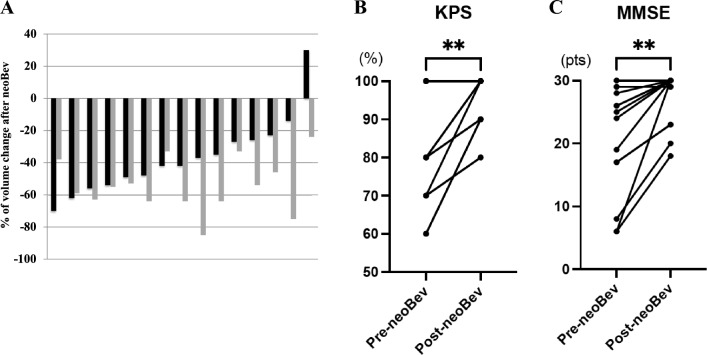
**A** Waterfall plot of tumor volume reduction after neoBev. The median reduction rates for T1CE (black bar) and FLAIR (gray bar) are 36.2% and -54.0%, respectively. **(B**, **C** Comparison of Karnofsky Performance Scale (KPS) (**B**) and Mini-Mental State Examination (MMSE) (**C**) before and after neoBev. Significant differences are apparent for both (KPS, ** p<0.0024; MMSE, ** p<0.0026; paired-t test)

In comparison between MRI within 14 days before registration (baseline) and within 7 days before resection, tumor volume was decreased by preoperative therapy in all but one case (Case 7) on T1CE, and in all cases on FLAIR (Fig. 1A, Supplementary Fig. 2). Mean tumor volume decrease rates on T1CE and FLAIR were − 36.2% and − 54.0%, respectively. Importantly, the decrease rate on T1CE did not correlate with that on FLAIR. Mean ADCs before and after preoperative therapy were similar, and no obvious trends toward changes in ADCs were noted with therapy (Supplementary Table 1).

KPS significantly improved with preoperative therapy, with a mean pre-therapy (within 5 days before registration) score of 83.3 and a mean post-therapy (within 7 days before resection) score of 94.7 (Fig. 1B; p < 0.0024; paired-t test). MMSE score also significantly improved after preoperative therapy, with a pre-therapy mean of 21.3 and a post-therapy mean of 27.5 (Fig. 1C; p < 0.0026; paired-t test).

### Survival analyses

**Fig. 2:**
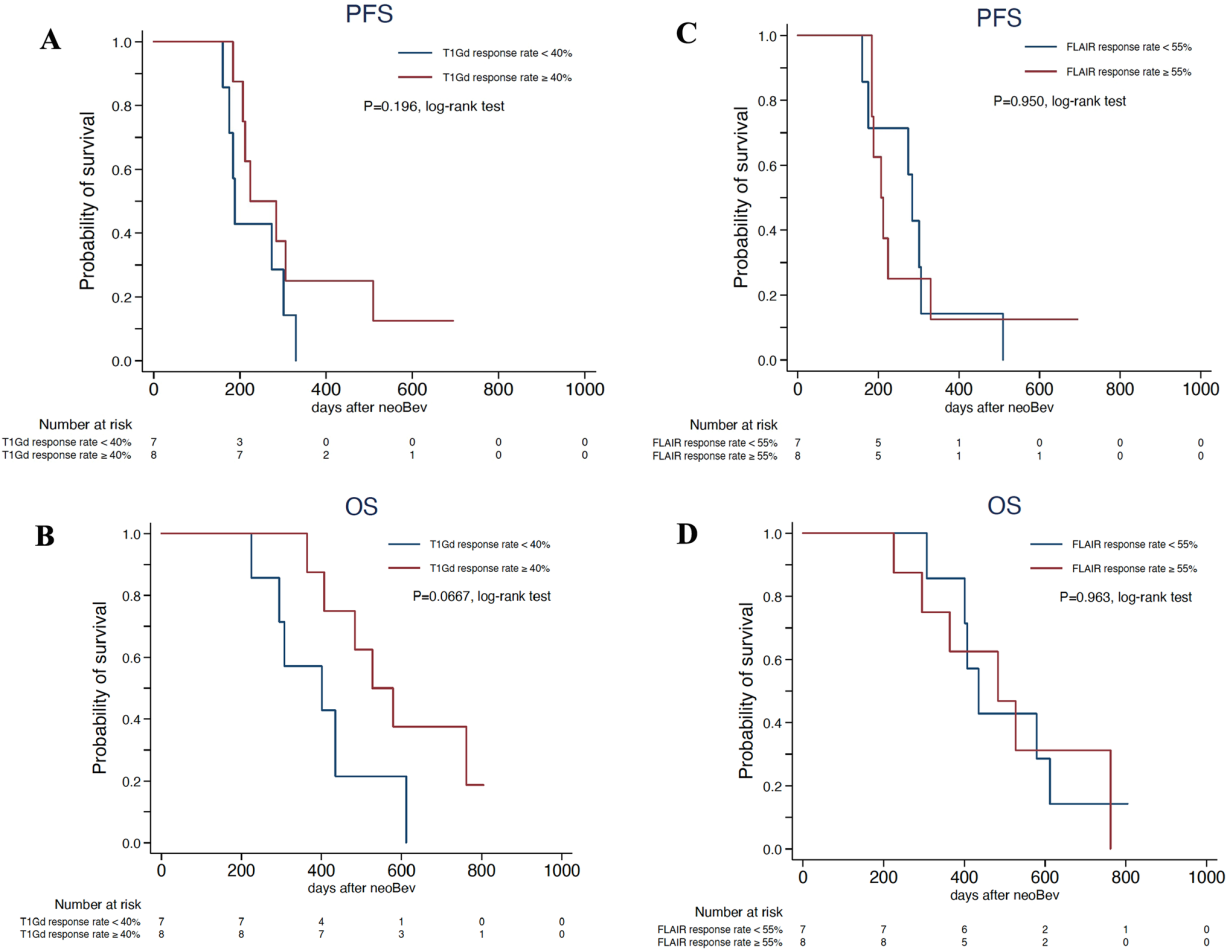
PFS and OS stratified by average neuroradiographic response rates on T1Gd **A**, **B** and FLAIR **C**, **D** after neoadjuvant Bev. T1CE good responders and poor responders are defined as neuroradiographic response rate on T1Gd ≥40% and <40%, respectively. In the same way, FLAIR GR and PR are defined as neuroradiographic response rate on T1CE ≥55% and <55%, respectively. **(A)** Median PFS for T1CE GR and PR. **B** Median OS for T1CE GR and PR. **C** Median PFS for FLAIR GR and PR. **D** Median OS for FLAIR GR and PR

Median PFS and OS in the present cohort were 9.5 months and 16.5 months, respectively. The 2-year OS rate was 20%. To determine whether any correlation existed between radiological response and patient survival, PFS and OS were compared between good responders (G-Res) and poor responders (P-Res) on each of T1CE and FLAIR images. G-Res and P-Res on T1CE were defined as showing radiological response rates of ≥ 40% and < 40% on T1CE based on the median response rate. In the same way, G-Res and P-Res on FLAIR were defined as radiological response rate of ≥ 55% and < 55% on FLAIR, respectively. Median PFS of G-Res and P-Res on T1CE were 11.0 months and 7.7 months, respectively (p = 0.196) (Fig. 2A). Median OS of G-Res and P-Res on T1CE were 19.8 months and 12.9 months, respectively (p = 0.0667) (Fig. 2B). In contrast, median PFS of G-Res and P-Res on FLAIR were 9.4 months and 9.6 months, respectively (p = 0.950) (Fig. 2C), and median OS were 16.0 months and 17.0 months, respectively (p = 0.963) (Fig. 2D).

### Adverse events

Three SAEs possibly related to preoperative Bev and TMZ were observed in 2 patients, comprising wound infection in one, postoperative hematoma in the resection cavity and thrombocytopenia in the other. In a patient with postoperative wound infection (Case 2), a carmustine wafer had been implanted in the resection cavity. Actually, the protocol was amended after this SAE with the addition of the instructions regarding wound handling (see Treatment in Methods). In the patient with a multifocal deep-seated tumor, postoperative hematoma in the resection cavity occurred immediately after partial resection performed on day 27 (Case 8). The hematoma was removed on the day of resection. The platelet count in this patient was 228,000/μL before administration of Bev and TMZ, 132,000/μL before resection surgery, and 30,000/μL immediately before surgery for hematoma removal. Fortunately, the patient fully recovered to her preoperative status immediately. The postoperative hemorrhage was likely attributable to residual tumor and insufficient hemostasis, and thrombocytopenia was considered mostly attributable to consumption of platelets during the resection surgery. However, the DSMC pointed out that the decrease in platelet count before resection could have been associated with preoperative TMZ.

### Illustrative cases

#### Case 6

**Fig. 3:**
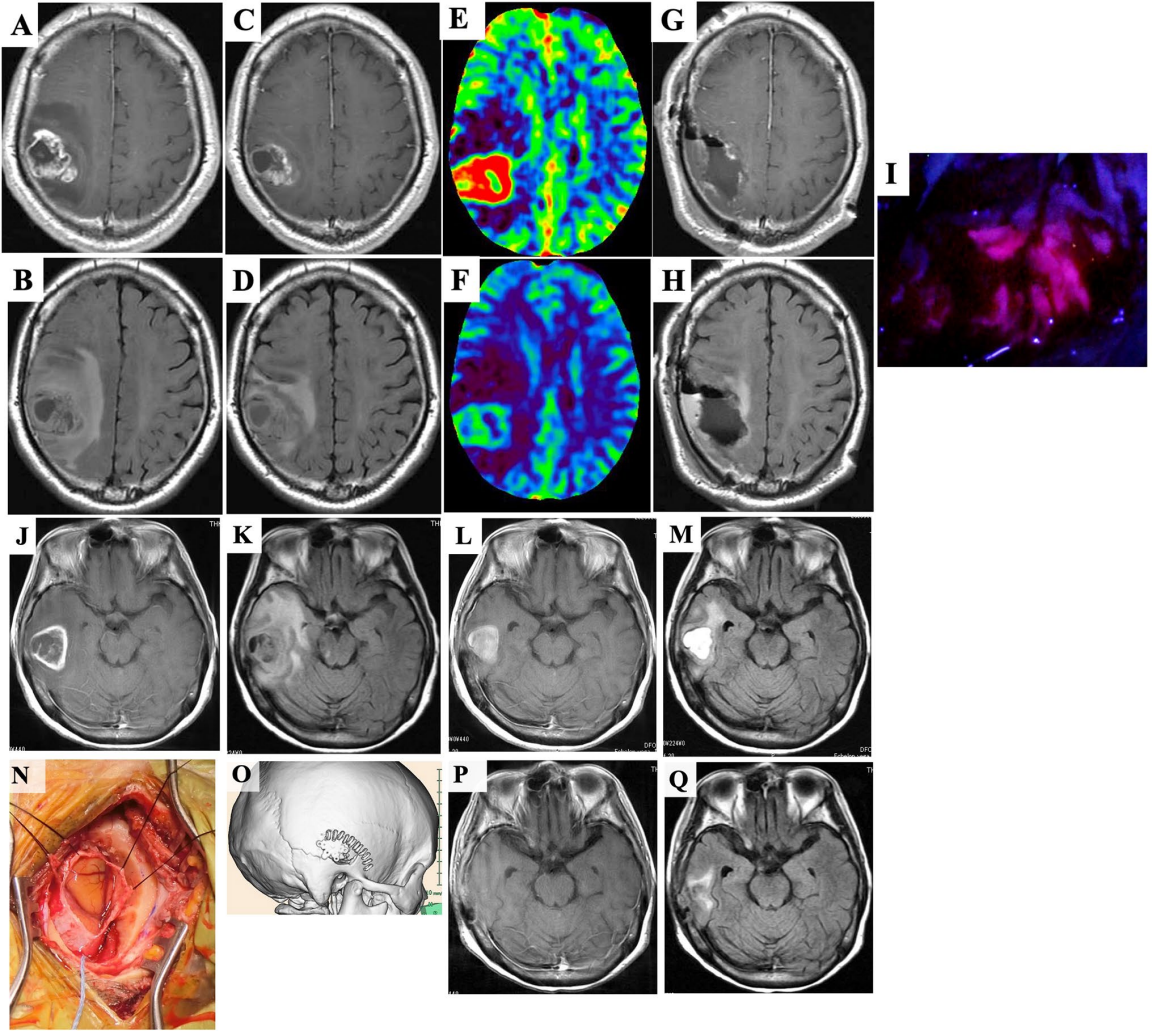
Illustrative Case 1 (Case 6). Preoperative MRI. Before preoperative neoadjuvant bevacizumab (neoBev), T1CE (**A**) and FLAIR (**B**) reveal an enhancing tumor with perifocal edema in the right middle temporal gyrus. Two weeks after neoBev, T1CE (**C**) and FLAIR (**D**) reveal decreases in both size of the enhancing lesion and perifocal edema (− 54% and − 55%, respectively). Perfusion CT demonstrates tumor blood volume (TBV) before (**E**) and after (**F**) neoBev. TBV is significantly decreased after neoBev. Postoperative T1CE (**G**) and FLAIR (**H**) show complete disappearance of the tumor. Intraoperative fluorescence diagnosis with 5-ALA, suggesting that undiminished protoporphyrin IX fluorescence by the preoperative neoBev therapy (**I**). Illustrative Case 2 (Case 15). Preoperative MRI. Before preoperative neoadjuvant bevacizumab (neoBev), T1CE (J) and FLAIR (K) reveal an enhancing tumor with perifocal edema in the right middle temporal gyrus. Two weeks after neoBev, T1CE (**L**) and FLAIR (**M**) reveal decreases in both size of the enhancing lesion and perifocal edema (− 37% and − 85%, respectively). Intraoperative image shows a yellowish, less vascular tumor in the right middle temporal gyrus removed through trephination (**N**). Postoperative three-dimensional CT shows removal of the tumor through the small cranial window (**O**). Postoperative T1CE (**P**) and FLAIR (**Q**) show complete disappearance of the tumor

A 70-year-old man presented with right clumsiness. MRI revealed a tumor in the right parietal lobe with an irregular, ring-like enhancing mass on T1CE with perifocal edema on FLAIR (Fig. 3A, B). After he received Bev and TMZ, preoperative MRI revealed that the tumor volume was reduced by 54% on T1CE and 55% on FLAIR (Fig. 3C, D). Tumor blood volume (TBV) was also dramatically reduced by preoperative Bev and TMZ compared with before the therapy (Fig. 3E, F). Since MMSE score improved from 24 to 30, awake surgery was planned. On day 21, he underwent supratotal resection of the tumor beyond the contrast-enhanced volume (Fig. 3G, H). Intraoperative fluorescence intensity with 5-ALA was maintained (Fig. 3I). The histopathological diagnosis was GB, IDH-wildtype. The postoperative clinical course was uneventful.

#### Case 15

A 74-year-old woman presented with headache. MRI revealed a tumor in the right middle temporal gyrus showing a ring-shaped enhanced mass on T1CE with perifocal edema on FLAIR (Fig. 3J, K). The tumor volume was reduced by 37% on T1CE and 85% on FLAIR by the preoperative Bev and TMZ (Fig. 3L, M). She underwent minimally invasive trephination surgery under local anesthesia on day 28 (Fig. 3N ~ Q). The histological diagnosis was GB, IDH-wildtype. She was discharged 3 days after the operation without neurological deficits.

## Discussion

### Rationale for the study protocol

The Food and Drug Administration in the United States provides a warning that use of Bev should be withheld within 28 days before and after major surgery to avoid wound-healing complications (WHC). Indeed, perioperative use of Bev could be associated with an increased risk of WHC, by the nature of inhibiting the angiogenesis essential to wound healing [12–15]. However, we have not previously experienced any WHC or other complications in 8 cases where tumor resection was performed within 28 days of Bev administration [7, 16]. We therefore considered preoperative use of Bev in the neoadjuvant setting as feasible. Given the half-life of Bev and postoperative peak level of VEGF [17], we proposed that resection surgery might be best performed 3–4 weeks after Bev administration to maximize the benefits and minimize the risks. TMZ was included to minimize the risk of tumor extension due to insufficient efficacy of Bev during the wait for surgery.

### Advantages of preoperative bev

The primary aim of neoadjuvant therapy is to reduce the size or extent of the tumor before surgery, to decrease the difficulty and morbidity of procedures and increase the probability of success. For example, the utility of alkylating agent chemotherapy for oligodendrogliomas in the neoadjuvant setting has been reported [18, 19]. Given the highest expression of VEGF in GB, preoperative use of Bev is expected to reduce tumor volume, tumor vascularity, and peritumoral edema, possibly leading to safer surgery with a reduced risk. Indeed, most tumors in the present study appeared less vascular and brain swelling was improved, rather than the prominent hypervascularity usually seen for GB.

Moreover, the reductions of tumor volume and perifocal edema by Bev could improve the preoperative PS of patients, as the most important prognostic factor in GB patients [20]. Indeed, both KPS and MMSE scores in the present study were significantly improved by a single injection of preoperative Bev, which was shown to contribute to a reduced surgical burden and improved preoperative PS in patients. As a result, preoperative Bev enabled awake surgery in 2 cases due to improvements in language function or limb weakness in a short period.

### Safety

At the planning of the study, we considered possible increases in WHC, perioperative hemorrhagic events, and thromboembolic events. Among the 15 cases, we encountered 1 case with CTCAE grade 3 wound dehiscence and infection, and 1 case with postoperative hematoma. In both cases, the patient fortunately recovered to their full preoperative status by salvage surgery. The former adverse event occurred in the case for which carmustine wafers were placed during tumor resection, and was likely associated with both Bev and carmustine wafer. Indeed, WHC was not seen in any of the 8 cases in our previous experience in which carmustine wafers were not placed, and in any of the other 14 cases in this study with the protocol amendment prohibiting the use of the wafer[7]. This latter event might have been associated with decreased platelets due to preoperative TMZ. Importantly, we have not encountered any preoperative hemorrhagic events and have not realized any problems in hemostasis during resection [7]. Moreover, the protoporphyrin IX fluorescence by 5-aminolevlinic acid was equivalent to that in usual practice. We therefore consider that preoperative Bev and subsequent tumor resection can be safely performed with careful wound handling.

In the present study, tumor enlargement during the waiting period was noted on T1CE in one case (Case 7). Given the decrease in tumor volume on FLAIR and weakened contrast enhancement on T1CE, some benefits were obtained with reduced vascularity and brain swelling. However, the possibility of ineffective therapy should be kept in mind.

A lack of histological verification can lead to incorrect diagnosis and subsequent ineffective therapy. In the present study, preoperative MRI findings with ring-like enhancement and extensive perifocal edema were among the eligibility criteria. In addition to this criterion, careful exclusion of other pathological conditions would minimize the risk of incorrect diagnosis. In fact, in the present study, all 15 tumors enrolled based on imaging findings were GB according to the WHO 2016 criteria.

### Radiological response and patient survival

Anti-VEGF therapies are known to often weaken contrast enhancement accompanied by decreases in hyperintensity volume on T2WI/FLAIR due to reduced vascular permeability. In the present study, the volume reduction rates on T1CE and FLAIR by preoperative Bev were not correlated, and the larger reduction rate on T1CE rather than FLAIR showed a tendency toward more prolonged OS. Because the volume reduction of FLAIR abnormality is likely to be susceptible to pseudoresponse, our results might provide evidence that tumor volume on T1CE is more reliable indicator of response to Bev that could be associated with patient OS, than that on FLAIR. Indeed, previous reports have supported our results, demonstrating that early progression on T1CE but not FLAIR after Bev-containing chemoradiotherapy offers a prognostic indicator for OS in recurrent and newly diagnosed GB [21, 22].

Previous reports have suggested decreases in ADC, relative cerebral blood volume, and uptake on positron emission tomography with [^18^F]-fluoromisonidazole after Bev administration as predictors of good response to Bev [23]. In the present study, consistent tendencies in ADCs were not seen before and after neoadjuvant Bev. Some cases showed an apparent decrease in TBV after preoperative Bev.

### Utility and perspectives of preoperative bev

As mentioned earlier, preoperative Bev would help decrease the difficulty and morbidity of tumor resection, not only by reducing tumor vascularity and edema, but also by improving KPS. We expect that preoperative Bev might prove particularly useful in the following situations: a bulky hypervascular tumor for which the feeding arteries cannot be intercepted during the early phase of surgery, such as tumor encasing sylvian middle cerebral arteries, and a hypervascular tumor with extensive edema in the eloquent area, for which preoperative Bev might provide conditions allowing more accurate functional monitoring. Moreover, preoperative Bev might be beneficial for improving the PS of patients suffering from early clinical deterioration before initial resection.

On the other hand, the initial response to one-shot Bev thus did not translate into patient survival in this exploratory phase II study. In this regard, the concerns might be that the tumor invading front does not withdraw despite the initial volume reduction on T1CE. Nonetheless, preoperative Bev might provide a greater likelihood of supratotal resection or FLAIRrectomy of GBs, and thus could contribute to better local control in those tumors [24, 25]. Further studies including preoperative Bev and subsequent FLAIRectomy might be warranted.

## Conclusions

The present study demonstrated that use of Bev in the neoadjuvant setting is safe as long as some instructions are followed to avoid WHC such as postponement of suture removal and withholding the use of carmustine wafer. A strategy of preoperative Bev and subsequent resection might be useful for preoperatively reducing tumor burden and improving patient PS in a subset of GBs.

### Supplementary Information

Below is the link to the electronic supplementary material.

Supplementary Figure 1 (DOCX 153 KB)—Treatment protocol for preoperative bevacizumab and temozolomide. An initial safety evaluation was made for the initial 7 cases. For these 7 cases, adverse events within 3 months of resection surgery were evaluated, with the study to be stopped and not proceed in the event of more than three non-hematological severe adverse events (SAEs) possibly related to preoperative Bev and TMZ, or two cases that did not undergo resection surgery due to reasons possibly related to preoperative Bev and TMZ. Safety evaluations for the initial 7 cases demonstrated 1 non-hematological SAE within 3 months of resection surgery possibly related to the preoperative therapy, as grade 3 wound infection in Neo-Bev-2 in which a carmustine wafer had been placed. Neither cancelation nor postponement of resection surgery was required for any of the 7 cases and resection surgeries were performed as scheduled. The data and safety monitoring committee therefore judged the study protocol as tolerable and approved enrollment of the additional 8 cases.CT, computed tomography; GBM, glioblastoma multiforme; RT, radiotherapy; TMZ, temozolomide.

Supplementary Figure 2 (DOCX 10612 KB)—T1-weighted contrast-enhanced (T1CE) and fluid attenuated inversion recovery (FLAIR) axial magnetic resonance imagings (MRI) from all patients in the present study. (A) Before neoadjuvant bevacizumab (Pre-NeoBev) on T1CE, (B) After neoadjuvant bevacizumab (Post-NeoBev) on T1CE, (C) Pre-NeoBev on FLAIR, and (D) Post-NeoBev on FLAIR.

Supplementary Table 1 (DOCX 21 KB)

## Abbreviations

ADC: apparent diffusion coefficient
ALA: aminolevulinic acid
AST: aspartate aminotransferase
ALT: alanine aminotransferase
Bev: bevacizumab
CT: computed tomography
DSMC: data and safety monitoring committee
DWI: diffusion-weighted imaging
FLAIR: fluid-attenuated inversion recovery
GB: glioblastoma
IDH: isocitrate dehydrogenase
KPS: Karnofsky Performance Scale
MMSE: Mini-Mental State Examination
MRI: magnetic resonance imaging
NAA/Cho: N-acetyl acid/choline
OS: overall survival
PFS: progression-free survival
PS: performance status
RT: radiotherapy
SAE: severe adverse event
T1CE: T1-weighted imaging with contrast enhancement
TBV: tumor blood volume
TME: tumor microenvironment
TMZ: temozolomide
VEGF: vascular endothelial growth factor
WHC: wound-healing complications
