# Supplementary material for: An exploratory prospective phase II study of preoperative neoadjuvant bevacizumab and temozolomide for newly diagnosed glioblastoma
Source: J Neurooncol. 2024 Jan 31;166(3):557–67. doi: 10.1007/s11060-023-04544-8 (PMC10876816; doi:10.1007/s11060-023-04544-8)
Supplement: Supplementary file 1 — Supplementary Figure 1 (DOCX 153 KB)—Treatment protocol for preoperative bevacizumab and temozolomide. An initial safety evaluation was made for the initial 7 cases. For these 7 cases, adverse events within 3 months of resection surgery were evaluated, with the study to be stopped and not proceed in the event of more than three non-hematological severe adverse events (SAEs) possibly related to preoperative Bev and TMZ, or two cases that did not undergo resection surgery due to reasons possibly related to preoperative Bev and TMZ. Safety evaluations for the initial 7 cases demonstrated 1 non-hematological SAE within 3 months of resection surgery possibly related to the preoperative therapy, as grade 3 wound infection in Neo-Bev-2 in which a carmustine wafer had been placed. Neither cancelation nor postponement of resection surgery was required for any of the 7 cases and resection surgeries were performed as scheduled. The data and safety monitoring committee therefore judged the study protocol as tolerable and approved enrollment of the additional 8 cases.CT, computed tomography; GBM, glioblastoma multiforme; RT, radiotherapy; TMZ, temozolomide. [file 11060_2023_4544_MOESM1_ESM.docx]

**Supplementary Figure 1**


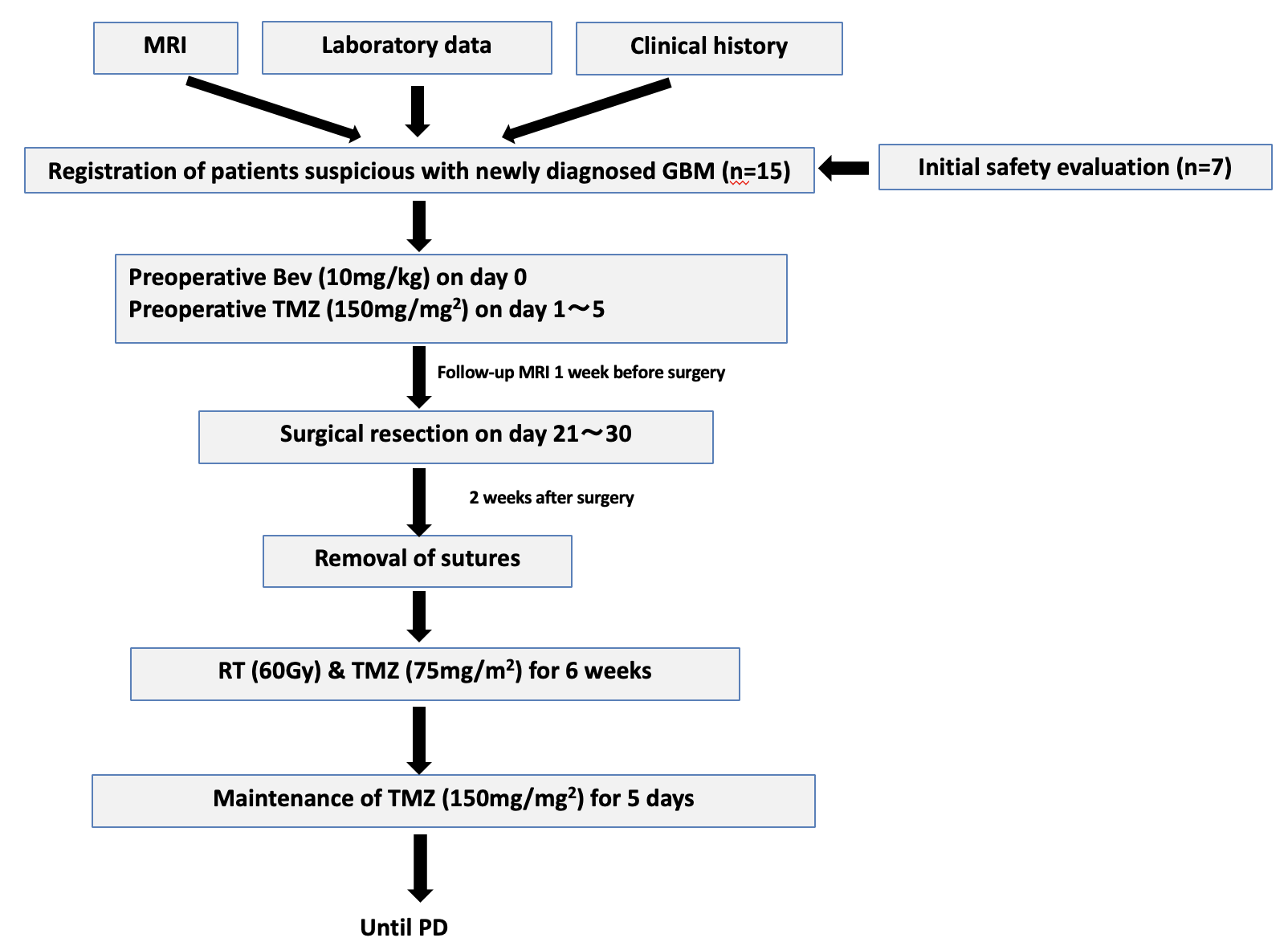


**Supplementary Figure 1:** Treatment protocol for preoperative bevacizumab and temozolomide. An initial safety evaluation was made for the initial 7 cases. For these 7 cases, adverse events within 3 months of resection surgery were evaluated, with the study to be stopped and not proceed in the event of more than three non-hematological severe adverse events (SAEs) possibly related to preoperative Bev and TMZ, or two cases that did not undergo resection surgery due to reasons possibly related to preoperative Bev and TMZ. Safety evaluations for the initial 7 cases demonstrated 1 non-hematological SAE within 3 months of resection surgery possibly related to the preoperative therapy, as grade 3 wound infection in Neo-Bev-2 in which a carmustine wafer had been placed. Neither cancelation nor postponement of resection surgery was required for any of the 7 cases and resection surgeries were performed as scheduled. The data and safety monitoring committee therefore judged the study protocol as tolerable and approved enrollment of the additional 8 cases.

CT, computed tomography; GBM, glioblastoma multiforme; RT, radiotherapy; TMZ, temozolomide.
