# Supplementary material for: An exploratory prospective phase II study of preoperative neoadjuvant bevacizumab and temozolomide for newly diagnosed glioblastoma
Source: J Neurooncol. 2024 Jan 31;166(3):557–67. doi: 10.1007/s11060-023-04544-8 (PMC10876816; doi:10.1007/s11060-023-04544-8)
Supplement: Supplementary file 2 — Supplementary Figure 2 (DOCX 10612 KB)—T1-weighted contrast-enhanced (T1CE) and fluid attenuated inversion recovery (FLAIR) axial magnetic resonance imagings (MRI) from all patients in the present study. (A) Before neoadjuvant bevacizumab (Pre-NeoBev) on T1CE, (B) After neoadjuvant bevacizumab (Post-NeoBev) on T1CE, (C) Pre-NeoBev on FLAIR, and (D) Post-NeoBev on FLAIR. [file 11060_2023_4544_MOESM2_ESM.docx]

**Supplementary Figure 2**


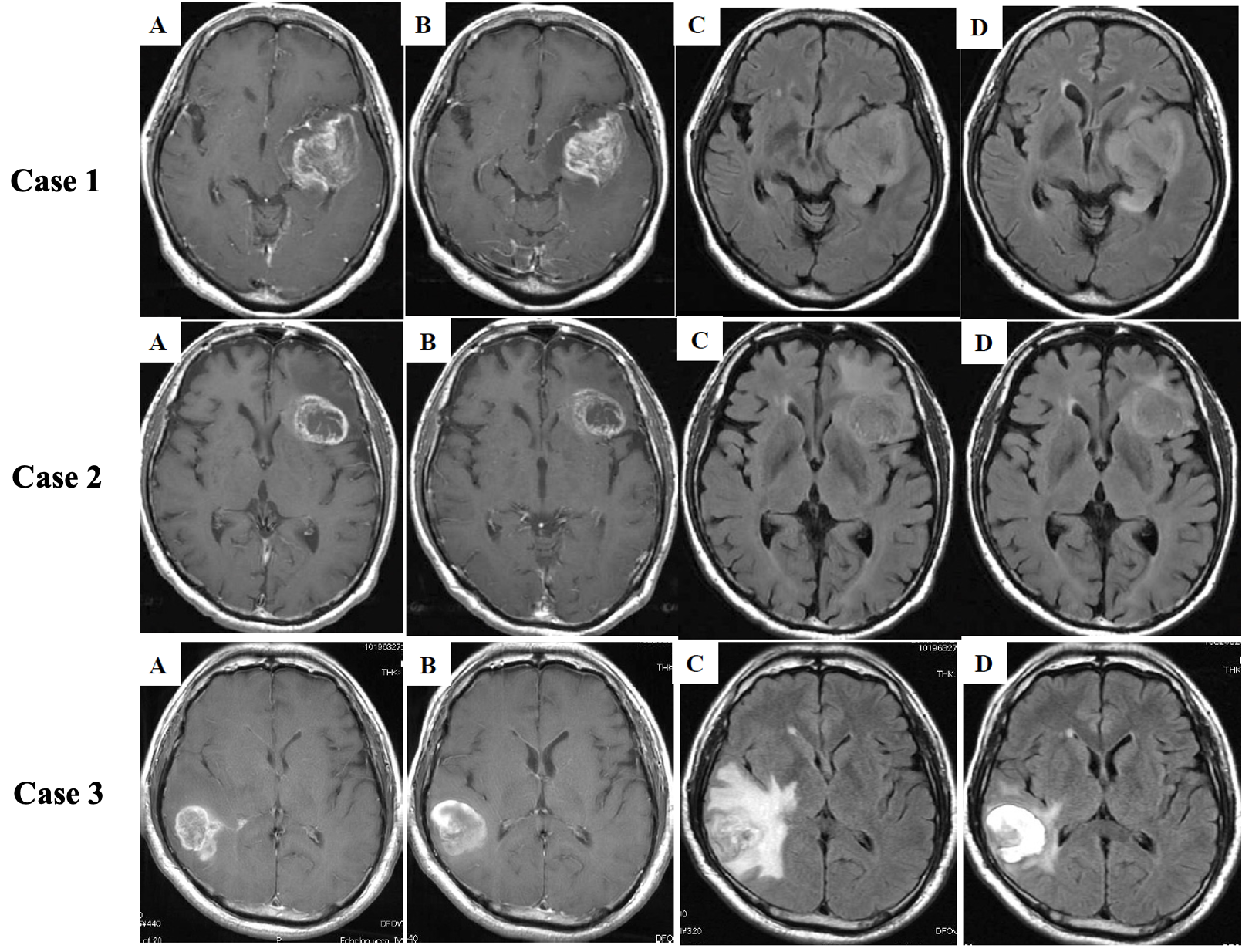


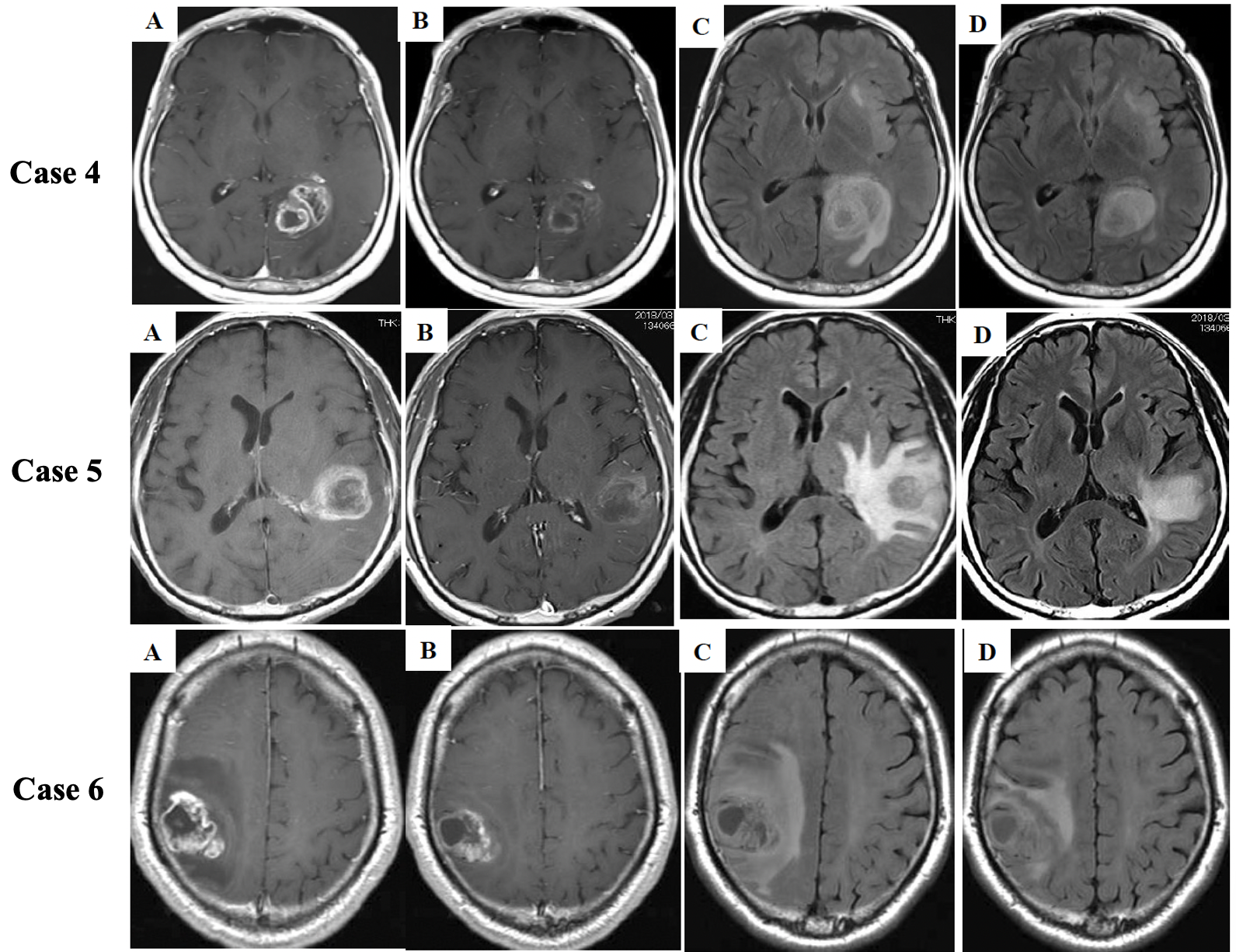


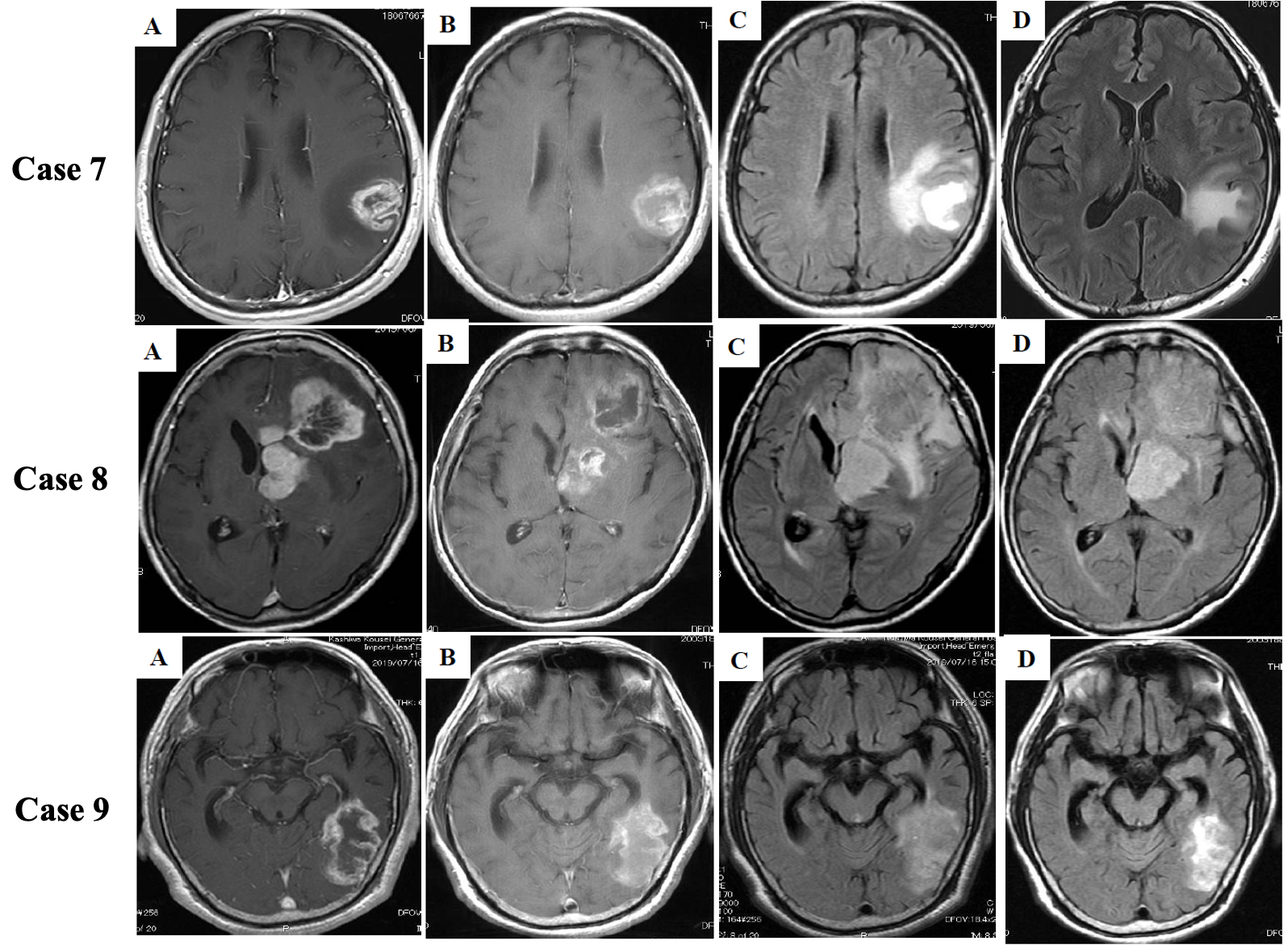


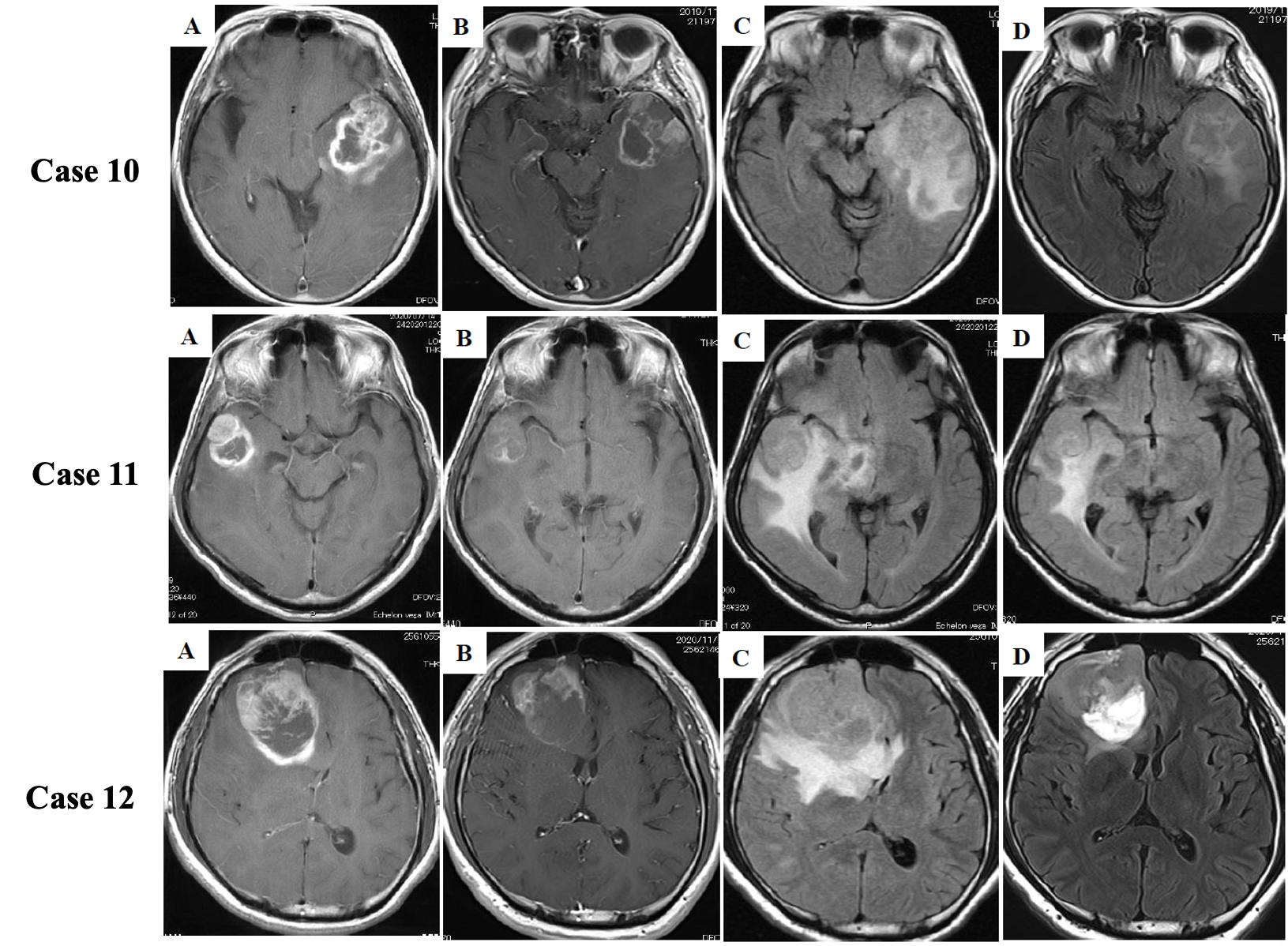


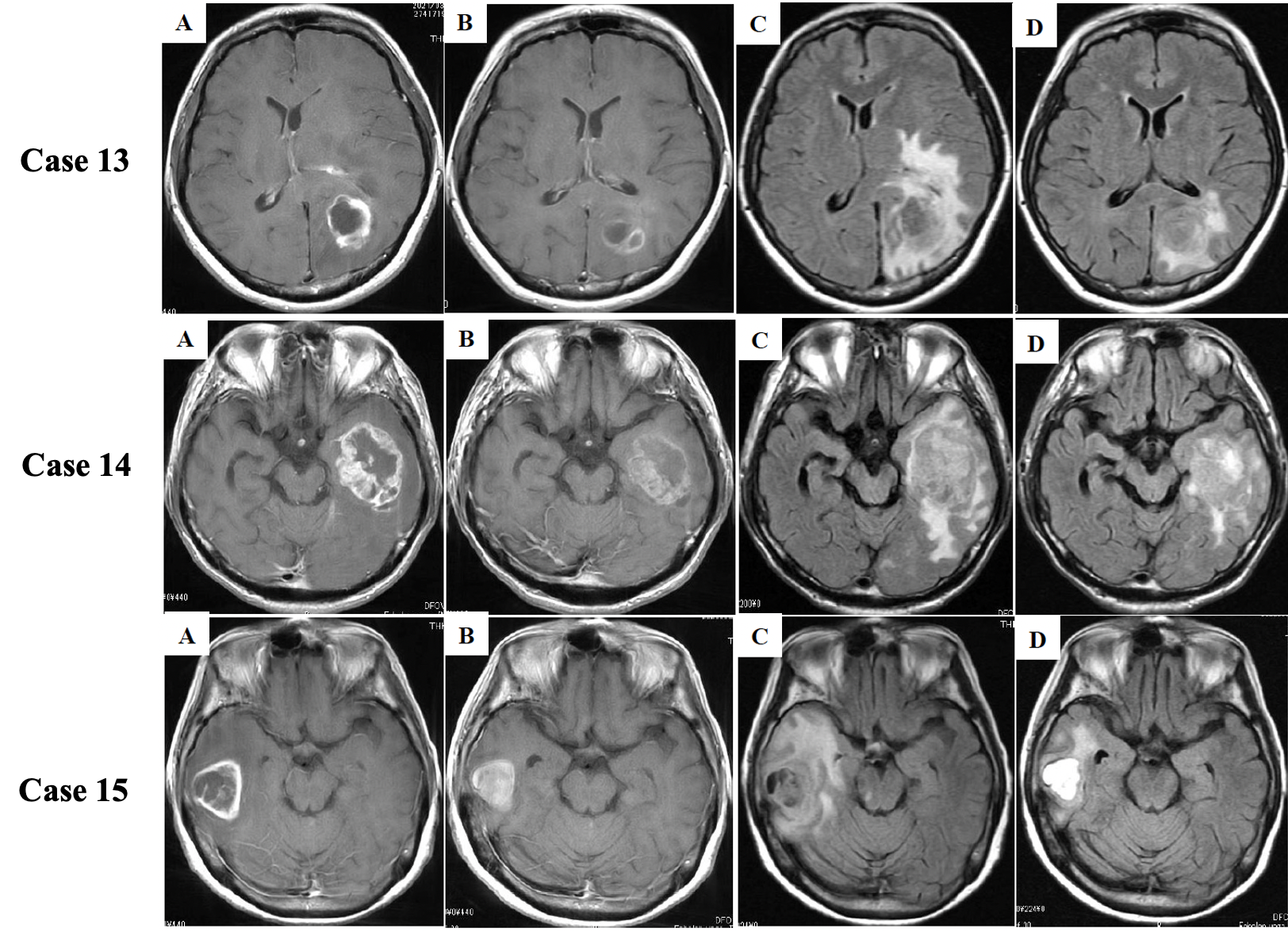


**Supplementary Figure 2:** T1-weighted contrast-enhanced (T1CE) and fluid attenuated inversion recovery (FLAIR) axial magnetic resonance imagings (MRI) from all patients (Case 1 to 15)

in the present study. **(A)** Before neoadjuvant bevacizumab (Pre-NeoBev) on T1CE, **(B)** After neoadjuvant bevacizumab (Post-NeoBev) on T1CE, **(C)** Pre-NeoBev on FLAIR, and **(D)** Post-NeoBev on FLAIR.
