# Supplementary material for: An exploratory prospective phase II study of preoperative neoadjuvant bevacizumab and temozolomide for newly diagnosed glioblastoma
Source: J Neurooncol. 2024 Jan 31;166(3):557–67. doi: 10.1007/s11060-023-04544-8 (PMC10876816; doi:10.1007/s11060-023-04544-8)
Supplement: Supplementary file 3 — Supplementary Table 1 (DOCX 21 KB) [file 11060_2023_4544_MOESM3_ESM.docx]

| **Supplementary Table 1** | | |  |  |  |  |  |  |  |  |  |  |  |  |  |  |  |  |  |  |  |  |  |
| --- | --- | --- | --- | --- | --- | --- | --- | --- | --- | --- | --- | --- | --- | --- | --- | --- | --- | --- | --- | --- | --- | --- | --- |
| **No.** | **age** | **sex** | **clinical Dx.** | **IDH-1 status** | **MIB-1 index (%)** | **Location** | **KPS before Bev** | **KPS after Bev** | **MMSE before bev** | **MMSE after bev** | **interval between neoBev to surgery (days)** | **pre- & postoperative steroid usage** | **Gliadel** | **awake surgery** | **5-ALA** | **EOR** | **PFS (months)** | **OS (months)** | **response rate after neoBev on T1CE (%)** | **response rate after neoBev on FLAIR (%)** | **ADC value before Bev** | **ADC value after Bev** | **ADC ratio before and after Bev** |
| **Case 1** | **57** | **F** | **GB** | **WT** | **30** | **Lt. temporal** | **80** | **100** | **26** | **30** | **23** | **not used** | **ー** | **ー** | **＋** | **GTR** | **5.8** | **13.4** | **-27** | **-33** | **3759.5** | **4302.9** | **0.9** |
| **Case 2** | **69** | **M** | **GB** | **WT** | **10** | **Lt. frontal** | **70** | **100** | **25** | **30** | **29** | **before (neoBev) and after surgery** | **＋** | **＋** | **＋** | **GTR** | **10.2** | **13.6** | **-49** | **-53** | **5076.0** | **4330.2** | **1.2** |
| **Case 3** | **50** | **M** | **GB** | **WT** | **20** | **Rt. temporal** | **100** | **100** | **30** | **30** | **21** | **before surgery (neoBev)** | **ー** | **ー** | **ー** | **GTR** | **10** | **14.5** | **-26** | **-54** | **122.7** | **66.2** | **1.9** |
| **Case 4** | **61** | **F** | **GB** | **WT** | **16** | **Lt. parietal** | **60** | **90** | **29** | **29** | **25** | **before surgery (neoBev)** | **ー** | **ー** | **＋** | **GTR** | **6.1** | **17.6** | **-62** | **-59** | **2421.0** | **1859.0** | **1.3** |
| **Case 5** | **63** | **M** | **GB** | **WT** | **30** | **Lt. temporal** | **100** | **100** | **29** | **29** | **21** | **before (neoBev) and after surgery** | **ー** | **ー** | **＋** | **GTR** | **6.9** | **16.1** | **-56** | **-63** | **136.9** | **1262.2** | **0.1** |
| **Case 6** | **70** | **M** | **GB** | **WT** | **30** | **Rt. parietal** | **80** | **100** | **24** | **30** | **21** | **not used** | **ー** | **＋** | **＋** | **GTR** | **7.5** | **25.4** | **-54** | **-55** | **3434.7** | **3258.0** | **1.1** |
| **Case 7** | **53** | **M** | **GB** | **WT** | **40** | **Lt. parietal** | **100** | **100** | **30** | **30** | **21** | **not used** | **ー** | **ー** | **＋** | **STR** | **9.1** | **20.4** | **30** | **-24** | **1318.4** | **157.9** | **8.3** |
| **Case 8** | **68** | **F** | **GB** | **WT** | **40** | **Lt. frontal, basal ganglia** | **60** | **90** | **6** | **18** | **26** | **not used** | **ー** | **ー** | **ー** | **PR** | **17** | **19.3** | **-42** | **-33** | **1315.8** | **137.9** | **9.5** |
| **Case 9** | **70** | **M** | **GB** | **WT** | **40** | **Lt. temporal** | **80** | **90** | **17** | **23** | **24** | **before (neoBev) and after surgery** | **ー** | **ー** | **＋** | **GTR** | **7.1** | **12.1** | **-42** | **-64** | **112.2** | **94.5** | **1.2** |
| **Case 10** | **72** | **F** | **GB** | **WT** | **80** | **Lt. temporal** | **70** | **80** | **6** | **30** | **28** | **not used** | **ー** | **ー** | **＋** | **STR** | **5.3** | **10.2** | **-23** | **-46** | **111.2** | **799.8** | **0.1** |
| **Case 11** | **69** | **M** | **GB** | **WT** | **30** | **Rt. temporal** | **100** | **100** | **17** | **23** | **21** | **not used** | **ー** | **ー** | **ー** | **GTR** | **9.5** | **27.7** | **-70** | **-38** | **87.9** | **76.7** | **1.1** |
| **Case 12** | **36** | **M** | **GB** | **mut** | **40** | **Rt. frontal** | **100** | **100** | **26** | **30** | **26** | **before surgery (neoBev)** | **ー** | **ー** | **ー** | **GTR** | **24** | **24.8** | **-48** | **-64** | **132.9** | **1260.9** | **0.1** |
| **Case 13** | **51** | **F** | **GB** | **WT** | **30** | **Lt. occipital** | **80** | **90** | **19** | **30** | **22** | **before surgery (neoBev)** | **ー** | **ー** | **＋** | **GTR** | **6.3** | **9.8** | **-14** | **-75** | **160.6** | **146.7** | **1.1** |
| **Case 14** | **70** | **M** | **GB** | **WT** | **25** | **Lt. temporal** | **70** | **80** | **8** | **20** | **22** | **not used** | **ー** | **ー** | **＋** | **GTR** | **6.3** | **7.5** | **-35** | **-64** | **128.5** | **102.1** | **1.3** |
| **Case 15** | **74** | **F** | **GB** | **WT** | **13** | **Rt. temporal** | **100** | **100** | **28** | **30** | **26** | **not used** | **ー** | **ー** | **ー** | **STR** | **11** | **14.7** | **-37** | **-85** | **128.6** | **83.0** | **1.5** |

**Supplementary Table 1:** Clinical data of patients.

ADC, apparent diffusion coefficient; 5-ALA, 5-aminolevlinic acid; Bev, bevacizumab; EOR, extent of resection; F, female; M, male; FLAIR, fluid-attenuated inversion recovery; GB, glioblastoma; GTR, gross total resection; KPS, Karnofsky Performance Scale; Lt, left; Rt, right; MMSE, Mini-Mental State Examination; OS, overall survival; PFS, progression-free survival; PR, partial resection; STR, subtotal resection; T1CE, T1-weighted imaging with contrast enhancement; WT, wild type.
